# Effect of aerobic exercise, slow deep breathing and mindfulness meditation on cortisol and glucose levels in women with type 2 diabetes mellitus: a randomized controlled trial

**DOI:** 10.3389/fphys.2023.1186546

**Published:** 2023-07-13

**Authors:** Hany Ezzat Obaya, Heba Ahmed Abdeen, Alae Ahmed Salem, Mai Ali Shehata, Monira I. Aldhahi, Taulant Muka, Elena Marques-Sule, Mona Mohamed Taha, Marwa Gaber, Hady Atef

**Affiliations:** ^1^ Department of Physical Therapy for Cardiovascular/Respiratory Disorder and Geriatrics, Faculty of Physical Therapy, Cairo University, Giza, Egypt; ^2^ Outpatients’ Clinic of Faculty of Physical Therapy, Cairo University, Giza, Egypt; ^3^ Department of Physical Therapy for Women’s Health, Faculty of Physical Therapy, Cairo University, Giza, Egypt; ^4^ Department of Rehabilitation Sciences, College of Health and Rehabilitation Sciences, Princess Nourah bint Abdulrahman University, Riyadh, Saudi Arabia; ^5^ Institute of Social and Preventive Medicine (ISPM), University of Bern, Bern, Switzerland; ^6^ Epistudia, Bern, Switzerland; ^7^ Physiotherapy in Motion, Multispecialty Research Group, Department of Physiotherapy, Faculty of Physiotherapy, University of Valencia, Valencia, Spain; ^8^ Medical Research Institute, Alexandria University, Alexandria, Egypt; ^9^ School of Allied Health Professions, Keele University, Staffordshire, United Kingdom

**Keywords:** aerobic training, breathing exercise, mindfulness meditation, serum cortisol concentrations, glucose

## Abstract

**Background:** Aerobic exercise combined with breathing exercise can be an integral part of diabetes mellitus treatment. This single-center, randomized, parallel-group study investigated the effect of the combination of aerobic exercise with slow deep breathing and mindfulness meditation on the glucose and cortisol levels of women with type 2 diabetes mellitus (T2DM).

**Materials and Methods:** Fifty-eight middle-aged women with T2DM (mean age: 45.67 ± 2.92 years) were randomly assigned to either the aerobic training group (AT: *n* = 29; mean age [46.1 ± 2.7 years]) or the aerobic exercise combined with slow deep breathing and mindfulness meditation (AT + DMM: *n* = 29; mean age [45.24 ± 3.14 years]). Aerobic exercise was performed at 60%–75% of the maximum heart rate. The women in each group were asked to perform the training three times weekly over a 6-week period. The duration of each session was 40 min for the AT group and 60 min for the AT + DMM group. The two groups were asked to perform aerobic exercise at 60%–75% of the maximum heart rate. Their fasting blood glucose (FBG) and serum cortisol levels were measured at the baseline and after the 6 weeks.

**Results:** Compared with the AT group, the group undertaking 6 weeks of aerobic training combined with slow, deep breathing exercises and mindfulness meditation showed significantly lower levels of FBG (*p* = 0.001) and cortisol levels (*p* = 0.01) than the AT group.

**Conclusion:** The addition of slow deep breathing and mindfulness meditation to aerobic exercise can better control the glucose and cortisol levels of women with T2DM and thereby improve their outcomes and decrease their cardiometabolic risk.

## 1 Introduction

Type 2 diabetes mellitus (T2DM) is becoming more prevalent globally, having increased from 8.3% in 2013% to 9.3% in 2019 (Chen-Ku et al., 2021). This increase has been greater in low-to middle-income countries than in high-income countries (Chen-Ku et al., 2021). In terms of gender, diabetic women have a higher risk of cardiovascular disease and mortality than diabetic men. (Emerging Risk Factors Collaboration et al., 2010). Therefore, understanding what kinds of interventions might improve a diabetes prognosis especially in women with T2DM, could help reduce sex disparities in diabetes (Peters et al., 2014; Glovaci et al., 2019).

Diabetes is classified as a metabolic disease and has been associated with stress. In fact, stress is considered a prominent factor among diabetes mellitus by stimulating the hypothalamus-pituitary-adrenal gland (HPA), the sympathetic nerve axis, as well as triggering parasympathetic nerve withdrawal, leading to increased circulating cortisol levels (Luo et al., 2018). From a prognostic perspective, diabetes is generally viewed as being more detrimental to women than men (Siddiqui et al., 2013). Additionally, research has shown that stress also has a negative impact on blood glucose levels, particularly in women (Kautzky-Willer et al., 2016). From a physiological perspective, chronic activation of the HPA axis makes it more difficult to control diabetes, as elevated levels of serum cortisol lead to higher levels of blood glucose and lipids in the bloodstream via gluconeogenesis (Dungan et al., 2009). Thus, control of cortisol levels using pharmacological and non-pharmacological approaches is an effective way to adjust glucose levels in diabetes patients (Salehi et al., 2019).

Physical exercise is among the key non-pharmacological interventions that modulate the HPA axis, which is a part of the stress response system (Stranahan et al., 2008). Aerobic exercise is an important component of a diabetes management plan as it can help improve insulin sensitivity, blood glucose control, and overall cardiovascular health. It is recommended that adults with diabetes engage in at least 150 min of moderate to vigorous aerobic exercise—such as brisk walking, cycling, swimming, or dancing weekly (Thompson et al., 2013). Aerobic exercise is an important component of a diabetes management plan, as it can help improve insulin sensitivity, blood glucose control, and overall cardiovascular health (Liguori and American College of Sports Medicine, 2020). In addition, exercise is proven to have clinical benefits for diabetics, such as improved insulin sensitivity, reduced glycosylated hemoglobin (HbA1c), and increased peak oxygen consumption (VO_2_ peak), which lowers the likelihood of diabetes. Exercise improves blood glucose control and mitigates cardiovascular risk factors and the overall mortality risk in patients with T2DM (Cannata et al., 2020).

Aside from aerobic exercise, other non-pharmaceutical interventions—such as diaphragmatic slow, deep breathing and mindfulness meditation training—have been found to influence stress levels. Diaphragmatic breathing has been reported to reduce the respiratory rate and maximizes the volume of gases in the blood (Ma et al., 2017). Thus, it follows that slow deep breathing can reduce stress, which in turn can lower blood sugar levels (Fiskin and Sahin, 2021; Mitsungnern et al., 2021). Numerous studies have shown that deep breathing exercises positively impact various T2DM risk factors, including hyperglycemia, stress, and anxiety (Ma et al., 2017; Dias et al., 2020; Pardede et al., 2020). Additionally, mindfulness meditation has been suggested as a helpful technique for people with anxiety, depression, and pain (Janssen et al., 2018). Mindfulness meditation involves sitting comfortably, focusing on breathing, and bringing the mind’s attention to the present moment without drifting into concerns about the past or the future (Hoge et al., 2018).

While the impact of breathing exercises, as well as aerobic exercise, on stress have been extensively studied, the combined effect of aerobic exercise, slow deep breathing, and mindfulness meditation has yet to be investigated. Therefore, this study investigated the effect of aerobic exercise; diaphragmatic slow, deep breathing, and mindfulness meditation on the glucose and cortisol levels of women with T2DM.

## 2 Materials and methods

### 2.1 Study design

A single-blinded, randomized-controlled trial was conducted; a 1:1 allocation ratio was used. The study was conducted from January to August 2021 and registered on clinicaltrials.gov (clinicaltials.gov identifier: NCT05231031). Ethical approval was obtained from the institutional review board before study commencement (PTREC/012/003210). The study followed the guidelines of the Declaration of Helsinki on the conduct of human research. The purpose and benefits of the study were explained to all participants and written informed consent was subsequently obtained.

### 2.2 Participants

Fifty-eight women diagnosed with T2DM were recruited from an outpatient clinic for this study. T2DM was diagnosed at a fasting blood sugar level of greater than or equal to 126 mg/dl (ElSayed et al., 2023). All the participants were prescreened to check that they were eligible to participate in the study. The inclusion criteria were that participants were adult women (≥18 years) with a low physical activity level as scored on the International Physical Activity Questionnaire (IPAQ), with T2DM for at least 5 years but were in a medically stable condition at the time of their enrollment and reported a moderate to high stress score f 14–40 points based on the Perceived Stress Scale (PSS) questionnaire (a valid and widely used instrument for measuring stress and the degree to which a person feels that a situation is stressful). (Simon, 2020). The exclusion criteria, which were ascertained by qualified health personnel, were an unstable medical condition; a sleeping disorder; an indication of receiving treatments for depression and anxiety; or having a musculoskeletal or neurological condition that might interfere with the implementation of the intervention or the assessment. Furthermore, any history of hepatic, renal, or vascular diseases; hypertension higher than 200/120 mmHg; or acute or chronic inflammatory disorders excluded participants from the study.

### 2.3 Randomization

**FIGURE 1:**
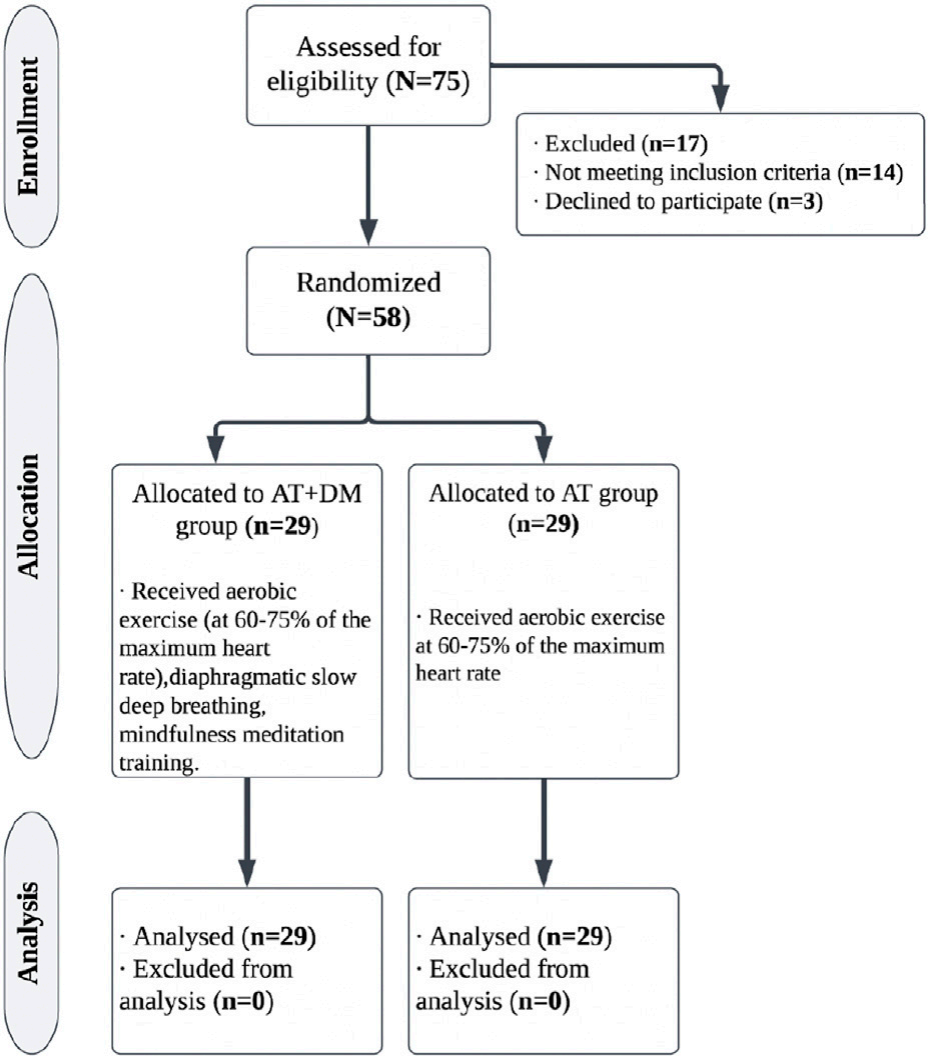
Experimental timeline.

In this study, participants were randomly assigned to one of two groups at 1:1 ratio. One group participating in aerobic training; slow, deep breathing; and mindfulness meditation (AT + DMM group [*n* = 29]), and a group only undertaking aerobic training (AT [*n* = 29]). This was achieved through a blinded research assistant opening sealed envelopes that contained a computer-generated randomization card and randomly allocating participants to each group (Figure 1).

### 2.4 Intervention

The aerobic training (AT) group engaged solely in aerobic exercise, whereas the AT + DMM group participated in aerobic exercise; diaphragmatic slow, deep breathing; and mindfulness meditation training. Both intervention approaches were conducted for three sessions per week over a duration of 6 weeks. The interventions were executed as follows:

#### 2.4.1 AT group

The participants in this group performed aerobic exercise only on a treadmill. The exercise was performed at an intensity of 60%–75% of the maximum heart rate, determined by a cardiopulmonary exercise test. Each participant was connected to ECG leads and instructed to walk on the treadmill, with their speed steadily increasing until volitional exhaustion. The test generally lasted between 8 and 12 min (Kosinski et al., 2019). The treadmill exercise program began with a 5-min warm-up phase in which each participant walked at a 0% grade for that period of time. Then, the treadmill workload (speed and grade) was calculated based on the metabolic equation of American College of Sports Medicine (ACSM) (Liguori and American College of Sports Medicine, 2020) and until the intensity of exercise reached 13–15 on the Borg Rating of Perceived Exertion (RPE) (Scherr et al., 2013). This was followed by a 5-min cooldown phase in which treadmill speed and grade were gradually reduced (Campbell et al., 2010). Therefore, the exercise session lasted for 40 min.

#### 2.4.2 AT + DMM group

Study participants in this group performed combination of aerobic exercise; diaphragmatic slow, deep breathing; and mindfulness meditation training in each session. The program of aerobic exercise was performed exactly as it was by the AT group; this was followed by diaphragmatic slow, deep breathing training in which participants were instructed to sit on a chair with the therapist standing beside them. The therapist guided the participants by instructing them to put one hand on their chest and the other hand on their abdomen and then asking them to take a normal breath. Then, they were asked to try deep breathing by breathing slowly through the nose, allowing the chest and lower belly to rise as the lungs were being filled. Participants were instructed to let the abdomen expand fully and then breathe out slowly through the mouth (Hazlett-Stevens and Craske, 2003). Exercise was performed for 6–10 repetitions per minute, with three to four repetition cycles of slow deep breathing. The total duration of the diaphragmatic slow deep training was 10 min. After that, the mindfulness meditation training was conducted in which participants were asked to sit comfortably and to relax for about 5 minutes. Participants sat in their chairs with a straight back, with their feet flat on the floor. They were asked to sit up straight so that their head and neck are in line with their spine while using a pillow behind the lower back and under the hips for added support (Sinha et al., 2018). Special emphasis was laid on breathing techniques practiced by each participant individually, and the same was checked on each subsequent visit**.** Music with a slow tempo and without lyrics was used to obtain more relaxation status for the participants (Umbrello et al., 2019). The total duration of the mindfulness meditation was 10 min. The exercise session lasted for a total of 60 min, encompassed a 5-min warm-up and a 5-min cooldown.

### 2.5 Outcome measures

Sociodemographic and clinical data were assessed at baseline by a trained researcher. At baseline, data related to participants’ age, weight, height, body mass index, and stress levels were measured. Stress levels were determined using the PSS. Scores on the PSS range from 0 to 40, with higher scores indicating higher levels of stress. According to Simon, (2020) stress levels can be categorized as low when scores fall between 0 and 13, moderate when scores range from 14 to 26, and high if scores range from 27 to 40. The questionnaire has been validated previously (Almadi et al., 2012). The IPAQ was used to assess participants’ physical activity levels. The IPAQ is a well-established, widely used self-reporting tool that assesses physical activity levels in various areas, placing results into categories of low, moderate, and high. The IPAQ has been validated in several studies and is recommended for measuring physical activity levels. Moreover, the questionnaire is simple and easy to administer and has good test-retest reliability (Bauman et al., 2011).

The primary outcome was serum cortisol levels. Total serum cortisol concentration was measured by employing the immunoassay method and using the Cortisol RIA KIT (Czech Republic). When collected at 8 a.m., the value ranged between 5.0 and 23.0 μg/dl (Pagana and Pagana, 2017; Yadegari et al., 2021).

The secondary outcome was the fasting blood glucose (FBG) levels. It was measured using the glucometer (MINDRAY BS 800, Bio-Medical Electronics Co., Shenzhen, China) at least 8 hours before which the participants were asked to refrain from eating and drinking. An ethylenediaminetetraacetic acid (EDTA) blood sample (2 ml in purple tube) was taken for glycemic control (HbA1c) using a kit manufactured by the Tianjin MD Pacific Technology Company of China.

### 2.6 Sample size calculation

Based on the results of the pilot study conducted at the beginning of the current study, the sample size was calculated before the start of the trial, with 10 subjects in each group. A *t*-test and G*Power statistical software (version 3.1.9.2; Franz Faul, Universitat Kiel, Germany) were used for calculation. The alpha level was set at 0.05; the effect size was 0.82; the *β* = 0.2. According to these specifications, the sample size was set at 50, and the number of dropouts was increased by 15%, resulting in a final number of 58.

### 2.7 Statistical analysis

The Shapiro–Wilk test was conducted to analyze the data normality. The data [age and BMI, stress total scale, HbA1c, cortisol, and fasting blood glucose (FBG) levels] were normally distributed, so parametric tests were performed. An unpaired *t*-test was used to analyze the demographic data (age, BMI, total stress score, and HbA1c). The outcome measures (the cortisol and FBG levels) were analyzed using two-way multivariate analysis of variance (MANOVA) to detect the effect of the treatments on all the measured variables and to elucidate the interaction between time and the treatments as well as the effect of time between the pre- and post-treatments. When MANOVA reported a significant effect, F-values dependent on the Wilks lambda test and the follow-up univariate ANOVA were used. Regarding the adjustment for multiple comparisons, the Tukey test was used to investigate the difference within each group and between the groups at the pre- and post-treatments. Partial eta squared (η^2^) was used to determine the size of the difference between the two groups. Additionally, the sizes of the treatment effects on cortisol and FBG within and between the groups were assessed using Cohen’s d test and were classified as large (d = 0.8), medium (d = 0.5), and small (d = 0.2). The data were analyzed using SPSS version 25 (IBM Corp., New York, United States of America), and the *p*-value was set at less than 0.05.

## 3 Results

### 3.1 Serum cortisol and fasting blood glucose levels

The 58 women in this study, who were aged 40–50 years with a mean age of 45.67 years, had HbA1c levels of 6.5%–7.5% and BMIs of 25–34.9 kg/m^2^. Table 1 shows their sociodemographic and clinical data at the baseline. No significant differences were found between the two groups at the baseline.

**TABLE 1:** Demographic and clinic characteristics of the study participants.

Variables	AT + DMM	At	*p*-value
	*n* = 29	*n* = 29	
Age (years)	45.24 ± 3.14	46.1 ± 2.7	0.26
BMI (kg/m^2^)	29.76 ± 3.02	30.19 ± 2.19	0.54
HbA1c %	6.86 ± 0.35	6.99 ± 0.3	0.84
Perceived Stress Scale-Total score	22.96 ± 2.77	22.82 ± 2.28	0.16
Physical activity level (MET-min**·**week^−1^)	325 ± 69	309 ± 55	0.33

Data are expressed as mean ± standard deviation. CI , confidence interval; BMI = body mass index; HbA1c) = Glycated hemoglobin.

Adherence to the treatment protocol was evaluated by dividing the number of completed treatment sessions that followed the protocol by the total number of scheduled sessions. The per-protocol treatment adherence rate was high, with almost 99% of the sessions adhering to the protocol. In the AE + DMM group, four participants missed one of the scheduled treatment sessions, whereas in the other group, five participants missed one session. However, there was no statistically significant difference between the adherence rates of the two groups (99.15% vs 98.94%, respectively; *p* = 0.936). Importantly, all the participants completed the remaining sessions in accordance with the protocol.

The MANOVA test reported the following statistically significant effects of the treatments: ʎ = 0.2, *p* = 0.04, f = 9.25, and η^2^ = 0.2. Moreover, the interaction between the treatments and the time had the following significant effects: ʎ = 0.68, *p* = 0.001, f = 12.48, and η^2^ = 0.3. Finally, there were significant effects on the time (pre- and post-treatments): ʎ = 0.07, *p* = 0.0001, f = 340.7, and *η* ^2^ = 0.92. Regarding the univariate ANOVA test, it showed statistically significant effects on the cortisol level (F = 6.96, *p* = 0.01, and *η* ^2^ = 0.11) and the FBG level (F = 12.86, *p* = 0.001, and *η* ^2^ = 0.18).

Table 2 shows the between-group analysis. There was no statistical difference between the cortisol and FBG levels of the experimental and control groups at the baseline, although there was a significant difference between the groups post-treatment. The mean difference between the cortisol levels of the groups in the post-treatment was 1.78 [95% confidence interval (CI): 3.12–0.4], and between the FBG levels, 5.89 (95% CI: 9.19–2.6). As shown in Table 2, the within-group analysis revealed statistically significant reductions in the cortisol and FBG levels in both groups. Specifically, the treatment effect size on the cortisol level was larger in the AE + SDB + MM group (mean difference = 5.49 and d = 2.07) than in the AE group (mean difference = 3.63 and d = 1.17). The difference between the effect sizes in the two groups was moderate (mean difference = 1.78, *p* = 0.01, and d = 0.69). Regarding the FBG level, the treatment effect size was larger in the AE + SDB + MM group (mean difference = 23.07 and d = 3.31) than in the AE group (mean difference = 15.8 and d = 1.78). The difference between the effect sizes in the two groups was large (mean difference = 5.89, *p* = 0.001, and d = 0.94).

**TABLE 2:** Comparison analysis for the fasting blood glucose and serum cortisol levels between and within the groups.

Parameters	Groups	Assessment timeline		95% CI	*p*-value**	Effect size (d)^a^
		Baseline	6-week			
Serum cortisol (nmol/L)	AT + DMM	18.08 ± 3.14	12.59 ± 2.17	4.45:6.52	0.0001	2.07
	AT	18.00 ± 3.34	14.37 ± 2.89	2.61:4.67	0.0001	1.17
	95% CI	0.06:0.1	3.12:0.4			
	*p*-value*	0.93	0.01			
	Effect size (d)^a^		0.69			
Fasting blood glucose (mg/dl)	AT + DMM	159.86 ± 8.73	136.79 ± 5.19	20.5:25.62	0.0001	3.31
	AT	158.48 ± 10.54	142.68 ± 7.16	13.23:18.35	0.0001	1.78
	95% CI	2.23:0.9	9.19:2.6			
	*p*-value*	0.59	0.001			
	Effect size (d)^a^		0.94			

Data are expressed as mean±standard deviation; CI, confidence interval. Statistically significant values of *p*-value <0.05 are showed in bold.

Denotes *p*-value between the group.

Denotes *p*-value within the group.

^a^ Treatment effect size (d): large if d = 0.8, medium if d = 0.5, small if d = 0.2.

## 4 Discussion

The results of this study revealed that combining slow deep breathing and mindfulness meditation with aerobic exercise significantly reduced the serum cortisol and FBG levels in women with T2DM than when only aerobic training was performed. To the best of our knowledge, this is the first study to explore the effectiveness of combined treatments that included aerobic training, slow deep breathing, and mindfulness meditation in stressed women with T2DM.

Regarding aerobic exercise, in this study, it decreased the serum cortisol levels by 20.16%. This result is consistent with that of study performed of de Souza et al. (2019) of a decrease in cortisol levels in response to daily moderate to high intensity aerobic exercise for five consecutive days, with a significant reduction from the first day of exercise to the fourth day (17.46 μg/dl and 8.39 μg/dl, respectively; *p* = 0.001). Our results further revealed that only performing aerobic exercise decreased FBG level by 9.97%. This result was supported by Pan et al. (2018), who compared the differences between different exercise training modalities for patients with T2DM, and found that aerobic exercise presented a more significantly improved the FBG levels (9.38% lower) than no exercise at all. This finding is aligned with the results of the participants in the aerobic exercise group in our study.

Breathing has been shown to be an effective way to manage stress and reduce cortisol levels. Previous studies have indicated a relationship between rhythmic breathing and the autonomous nervous system in the healthy controls and that such relationship often deteriorated with diabetes (Rivera et al., 2016). In this study, the addition of slow deep breathing and mindfulness meditation to aerobic exercise reduced the cortisol levels. There is growing evidence that rhythmic breathing can have a positive effect on the autonomic nervous system, particularly in individuals with diabetes. This is in line with a study by Ma et al. (2017) in which the cortisol levels significantly decreased after 20 sessions of diaphragmatic breathing, as the breathing activated the parasympathetic nervous system and reduced the sympathetic reaction, which can slow down the heart rate and decrease blood pressure, and, in turn, can help reduce muscle tension and promote relaxation. In addition, Örün et al. (2021) found a significant decrease in cortisol levels after an acute breathing exercise session. Moreover, in line with our findings, Schein et al. (Schein et al., 2020) concluded that breathing exercise significantly decreased FBG in T2DM patients. Likewise, Hedge et al. (2012) noted that breathing exercises could be incorporated in standard care as an add-on therapy to improve the glycemic parameters in T2DM patients. On the other hand, as Khanum et al. (2019) concluded that diaphragmatic breathing alone cannot maintain a normal blood sugar level among T2DM patients. Therefore, it is possible that a combined therapy approach that targets both the endocrine and autonomic nervous systems may have a synergistic effect and be more effective than diaphragmatic breathing exercises alone in maintaining normal blood sugar levels and cortisol levels in individuals with T2DM. In relation to mindfulness meditation, similar to our results, previous studies supported the significant effect of this technique on decreasing stress, anxiety and depression, and cortisol levels (Caplin et al., 2021; Dalpatadu et al., 2022). However, more research is needed to determine the most effective combination of therapies for managing both FBG and cortisol levels in individuals with T2DM.

To sum up, the combination of aerobic exercise, slow deep breathing, and mindfulness meditation in this study showed a significant reduction in cortisol and FBG levels by 30.29% and 14.54%, respectively. Our results further showed that the addition of other stress-controlling therapies, such as slow deep breathing and mindfulness meditation, may magnify the results, as seen in the intervention group. Thus, the combination of these techniques with traditional aerobic exercise may improve the control of blood glucose levels, which may enhance overall quality of life.

This study had some limitations. First, our findings may be generalized only to stressed women with T2DM. It would be prudent to investigate larger sample sizes of both genders in future studies to properly extrapolate the findings to the general population. Second, HbA1c was not measured because of the short duration of the intervention (6 weeks), which hindered our ability to detect the changes that might happen with HbA1c. Third, in this study, only a short-term assessment was conducted, although the importance of long-term assessments should be highlighted. Fourth, patient-reported outcomes could have been used as tools for exploring additional variables related to stress.

This study also had some strengths, though. First, it offered evidence of the effectiveness of a combined treatment of aerobic exercise, slow deep breathing, and mindfulness meditation, which was not commonly used in previous studies. In fact, scarce evidence is seen in this regard, and this is the first study that explored the effects of the three above-mentioned techniques in stressed women with T2DM.

## 5 Conclusion

These results indicate that the addition of slow deep breathing and mindfulness meditation to aerobic exercise can better control stress and glucose levels in women with T2DM, which will improve their outcomes and reduce their cardiometabolic risks. Also, as diabetes may have a worse prognosis in women and as the incidence of stress seems higher in women, future studies are needed to investigate the feasibility of employing additional techniques to improve stress and glucose levels in women. Thus, slow deep breathing and mindfulness meditation may be added to aerobic exercise as potentially useful components of the T2DM management program for stressed women.

## Data Availability

The original contributions presented in the study are included in the article/supplementary material, further inquiries can be directed to the corresponding author.
